# Establishment of oral squamous cell carcinoma cell line and magnetic bead-based isolation and characterization of its CD90/CD44 subpopulations

**DOI:** 10.18632/oncotarget.19914

**Published:** 2017-08-03

**Authors:** Marketa Svobodova, Martina Raudenska, Jaromir Gumulec, Jan Balvan, Michaela Fojtu, Monika Kratochvilova, Hana Polanska, Zuzana Horakova, Rom Kostrica, Petr Babula, Zbynek Heger, Michal Masarik

**Affiliations:** ^1^ Department of Physiology, Faculty of Medicine, Masaryk University, CZ-62500 Brno, Czech Republic; ^2^ Department of Pathological Physiology, Faculty of Medicine, Masaryk University, CZ-62500 Brno, Czech Republic; ^3^ Central European Institute of Technology, Brno University of Technology, CZ-61600 Brno, Czech Republic; ^4^ Department of Otorhinolaryngology and Head and Neck Surgery, St. Anne’s Faculty Hospital, CZ-65691 Brno, Czech Republic; ^5^ Department of Chemistry and Biochemistry, Mendel University in Brno, CZ-61300 Brno, Czech Republic

**Keywords:** head and neck neoplasms, coculture techniques, cell line, tumor, carcinoma

## Abstract

In this study, we describe the establishment of the human papillomavirus 18-positive, stage II, grade 1, T2N0M0 head and neck tumor primary cell line derived from oral squamous cell carcinoma of a non-smoking patient by using two different protocols. Furthermore, a preparation of subpopulations derived from this primary cell line according to the cluster of differentiation molecules CD44/CD90 status using magnetic bead-based separation and their characterization was performed. Impedance-based real-time cell analysis, enzyme-linked immunsorbant assay (ELISA), wound-healing assay, flow-cytometry, gene expression analysis, and MTT assay were used to characterize these four subpopulations (CD44^+^/CD90^−^, CD44^−^/CD90^−^, CD44^+^/CD90^+^, CD44^−^/CD90^−^). We optimised methodics for establishement of primary cell lines derived from oral squamous cell carcinoma tissue samples and subsequent separation of mesenchymal (CD90^+^) and epithelial (CD90^−^) types of tumorous cells. Primary cell line prepared by using trypsin proteolysis was more viable than the one prepared by using collagenase. According to our results, CD90 separation is a necessary step in preparation of permanent tumor-tissue derived cell lines. Based on the wound-healing assay, CD44^+^ cells exhibited stronger migratory capacity than CD44^−^ subpopulations. CD44^+^ subpopulations had also significantly higher expression of *BIRC5* and *SOX2*, lower expression of *FLT1* and *IL6*, and higher levels of basal autophagy compared to CD44^−^ subpopulations. Furthermore, co-cultivation experiments revealed that CD44^−^/CD90^+^ cells supported growth of epithelial tumor cells (CD44^+^/CD90^−^). On the contrary, factors released by CD44^+^/CD90^+^ type of cells seem to have rather inhibiting effect. The most cisplatin-resistant subpopulation with the shortest doubling time was CD44^−^/CD90^+^, but this subpopulation had a low migratory capacity.

## INTRODUCTION

Head and neck squamous cell carcinoma (HNSCC) is the sixth most frequent cancer worldwide. Regardless of advances in diagnostic methods and therapy, survival of HNSCC remains almost unchanged with treatment resistance and metastases being the most important indicator of adverse outcome [1]. A recently disclosed significant problem is the distinct cellular heterogeneity in the HNSCC tumor tissues that may contribute to formation of metastases or treatment resistance [2]. Recent data suggests that many potential cancer stem cell (CSC) markers are differentially expressed in different subpopulations of cells derived from a particular tumor [3]. One of the relevant characteristics of HNSCC subpopulations is the selective expression of surface receptors. In the context of HNSCC, CD44 and CD90 molecules are extensively discussed [4–6].

Surface glycoprotein CD44 is involved in cell-cell interactions, cell migration, and adhesiveness [7]. CD44 receptors are connected to the signalling cascade of EGFR and the PI3K/Akt and thus can significantly affect tumor progression [8]. The CD44^+^ phenotype is associated with head and neck, prostate, pancreatic, and breast cancer-initiator cells [9]. It has been also revealed that CD44 is important for metastasis as demonstrated on non-metastatic rat glioma cells. Those cells obtained metastatic ability when CD44 was over-expressed [10]. It was revealed that HNSCC cells positive for CD44 have the ability to produce tumors in immunocompromised mice. CD44^+^ cells are therefore often referred to as the CSCs [11, 12]. On the other hand, Lim et al. study puts the use of CD44 as a CSCs marker into question as they observed that both CD44+ and CD44− cells extracted from squamospheres are able to regenerate these spheres [13]. Nevertheless, CD44+ cancer cell population in primary HNSCC is comprised of less than 10% of bulk tumor [14] and HNSCC-driven squamospheres possessed enriched CD44+ cell population (53%) [13]. Furthermore, reduced CD44 expression resulted in a decreased proliferation and in an altered morphology of colonies suggesting a loss of stem cell character [15]. Moreover, CD44 expressed on cancer-associated fibroblasts (CAFs) seems to support the stemness and resistance of neighbouring malignant cells [16].

CD90 (also called THY1) was identified in the thymus as a T-cell differentiation and maturation marker [17], nevertheless human fibroblasts and cancer stromal cells also express abundant CD90 on their surface [18]. Immunohistochemical analysis showed overexpression of CD90 in cancer-associated stroma compared with non-cancer tissue stroma [17]. Furthermore, frequency of CD90-positive cells in HNSCC directly correlates with tumor volume [19]. Moreover, mesenchymal marker CD90 expressed on epithelial cells could be a marker of epithelial-mesenchymal transition (EMT) [4]. Lu et al. also suggested that CD90 could serve as an anchor used by carcinoma stem cells to attach tumor-associated monocytes and macrophages [20].

In this study, we describe the establishment of the head and neck tumor primary cell line derived from oral squamous cell carcinoma in a HPV18-positive, non-smoking patient (stage II, grade 1, T2N0M0) by using two different protocols. Furthermore, a preparation of subpopulations derived from this primary cell line according to the CD44/CD90 status using magnetic bead-based separation and their characterization was performed.

We also present an easy and cheap method for evaluation of the effects of factors released by distinct subpopulations in tumor tissue on cancer cell growth. In this study, we do not aspire to draw general principles of cancerogenesis and cellular interactions in tumor tissue. We would rather offer interesting methodics, procedures, and a new approach for cancer research.

## RESULTS

### Clinico-pathological characteristics of patient

The primary cell line was derived from tumor tissue taken from localized oral squamous cell carcinoma in a HPV^+^ (HPV16^−^; HPV18^+^), non-smoking male-patient (T2N0M0, stage II, grade I). The patient was at the age of 57 with BMI=22, had no previous history of tumors, no diabetes mellitus, hypertension, heart ischemia, or chronic kidney disease. This patient has also not undergone stroke or myocardial infarction. This patient did not receive preoperative radiotherapy or chemotherapy. Patient achieved a complete remission after surgery.

### Primary cell line establishment and characterization

The cell line was prepared by using two protocols; See the section Primary culture establishing and culture conditions. Primary cell line prepared according to the Protocol 2 was not as viable and the cell line designated 132P1 was successfully established according to the Protocol 1 (for details see “Primary culture establishing and culture conditions” section). The growth curve for the primary cell line 132P1 was as follows: lag phase 87 h, exponential phase 25 h, and plateau phase after the 112^th^ h. The doubling time was 22 h (Figure 2B). In our established primary cell line, other subpopulations were gradually overgrown by CD90^+^ cells (Figure 2A).

**Figure 1:**
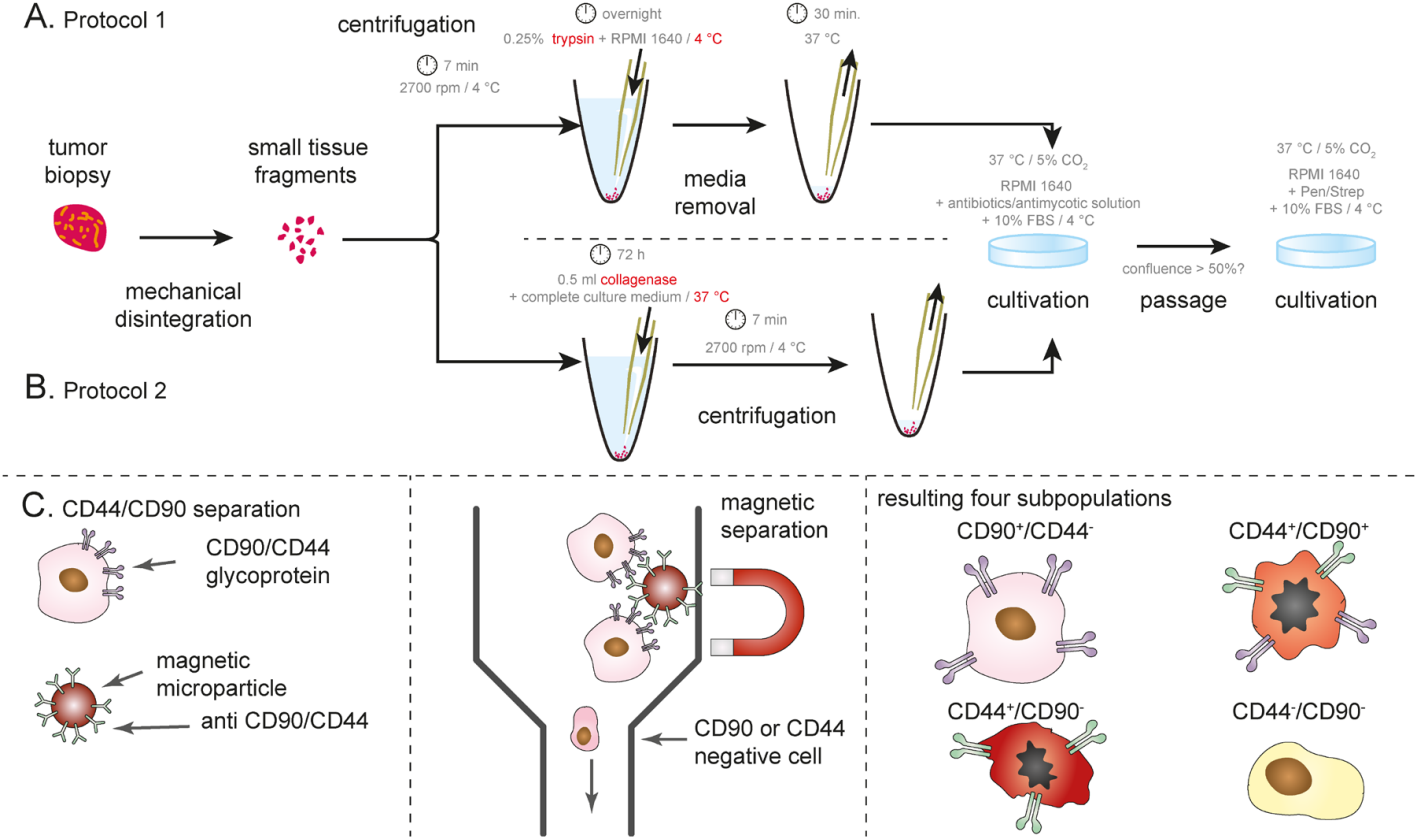
Schematic depiction of isolation protocol **(A)** Protocol 1 using trypsin, **(B)** collagenase-based isolation protocol. **(C)** Magnetic bead-based separation according CD44/CD90 surface antigens. Both positive- and negative-separation was utilized in order to obtain CD44/CD90 -positive and -negative cells. For details, see Materials and Methods.

**Figure 2:**
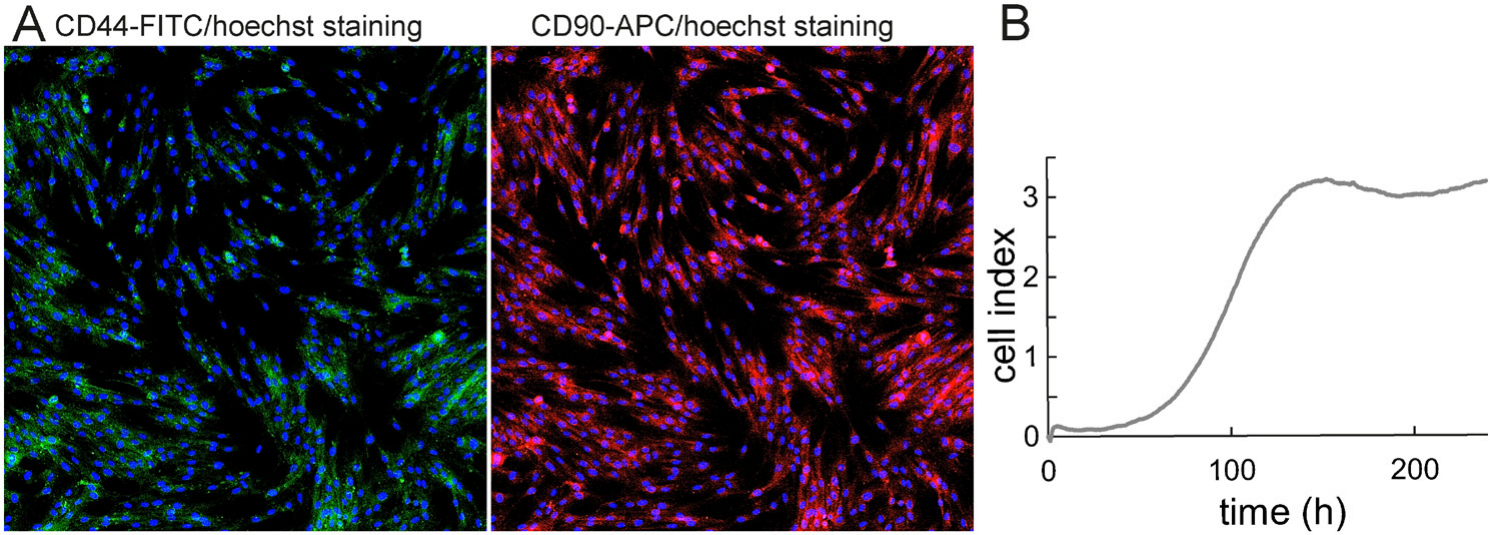
Fluorescence microscopy and growth curve of 132P1 unseparated primoculture **(A)** CD44 and CD90 staining of the unseparated cell line 132P1 after passage 15. Fluorescent staining revealed prevailing representation of CD90^+^ cells; 10× magnification. **(B)** Growth profile of unseparated cell line 132P1 using real-time cell analyser demonstrated as a dimensionless unit “cell index”.

### Morphology, growth and migratory characteristics of subpopulations

**Figure 3:**
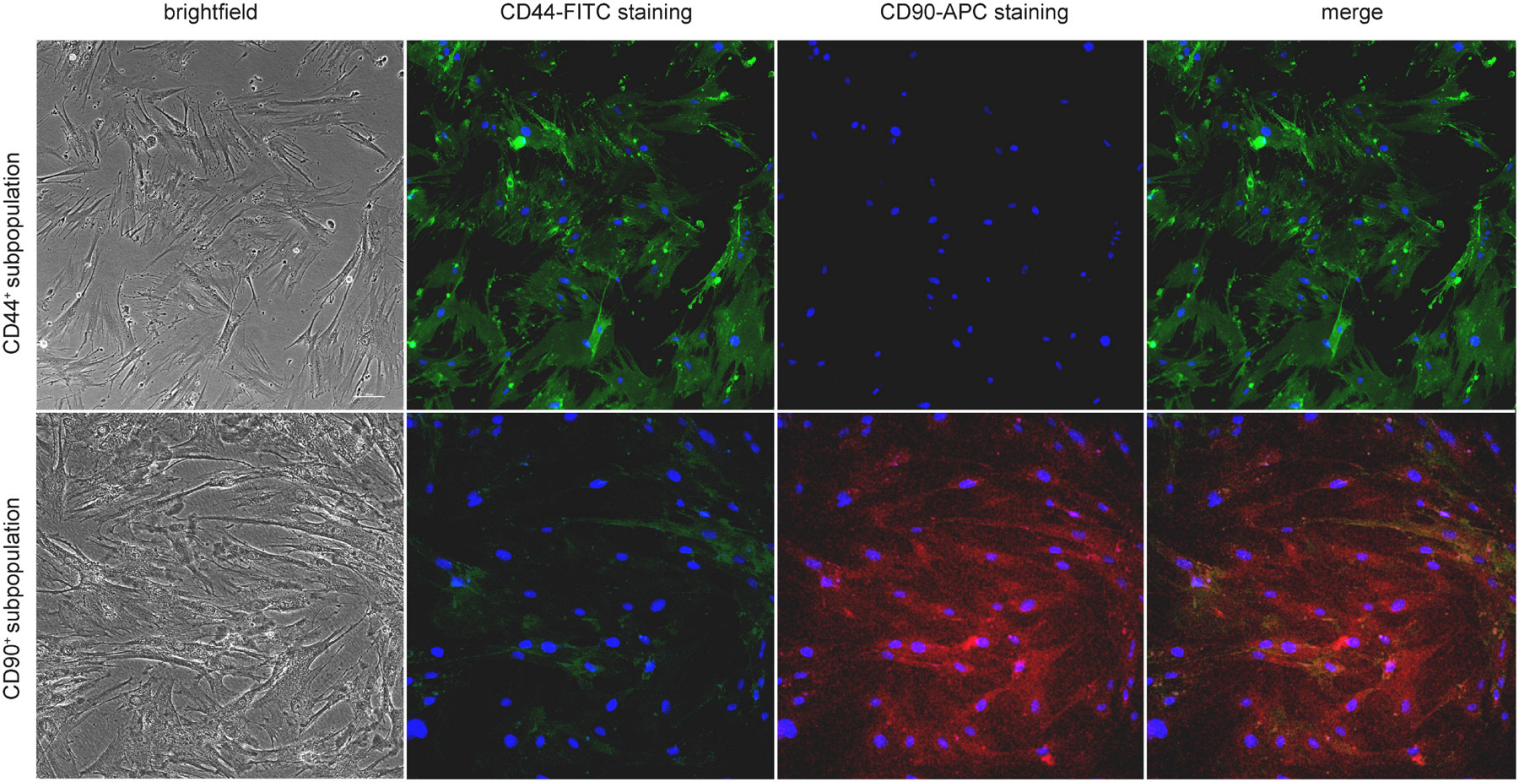
Fluorescent stainging of CD44 and CD90 in separated subpopulations Fluorescence confirms specificity of the sorting protocol; 20 × magnification.

In the next step, the primary cell line 132P1 was separated according to the CD44 and CD90 status (CD44^+^/CD90^−^, CD44^−^/CD90^−^, CD44^+^/CD90^+^, CD44^−^/CD90^+^). Separation according to expression of CD44 and CD90 molecules was verified by CD44-FITC/CD90-APC fluorescent staining (see Figure 3). After separation of four subpopulations, all four types of subpopulations were able to grow long-term in cell culture (18 passages). Nevertheless, after 18th h and 20th h passage, respectively, CD44^−^/CD90^−^ and CD44^−^/CD90^+^ subpopulations have slowed down their growth extremely and nearly stopped dividing, which was not observed in CD44^+^ subpopulations.

**Figure 4:**
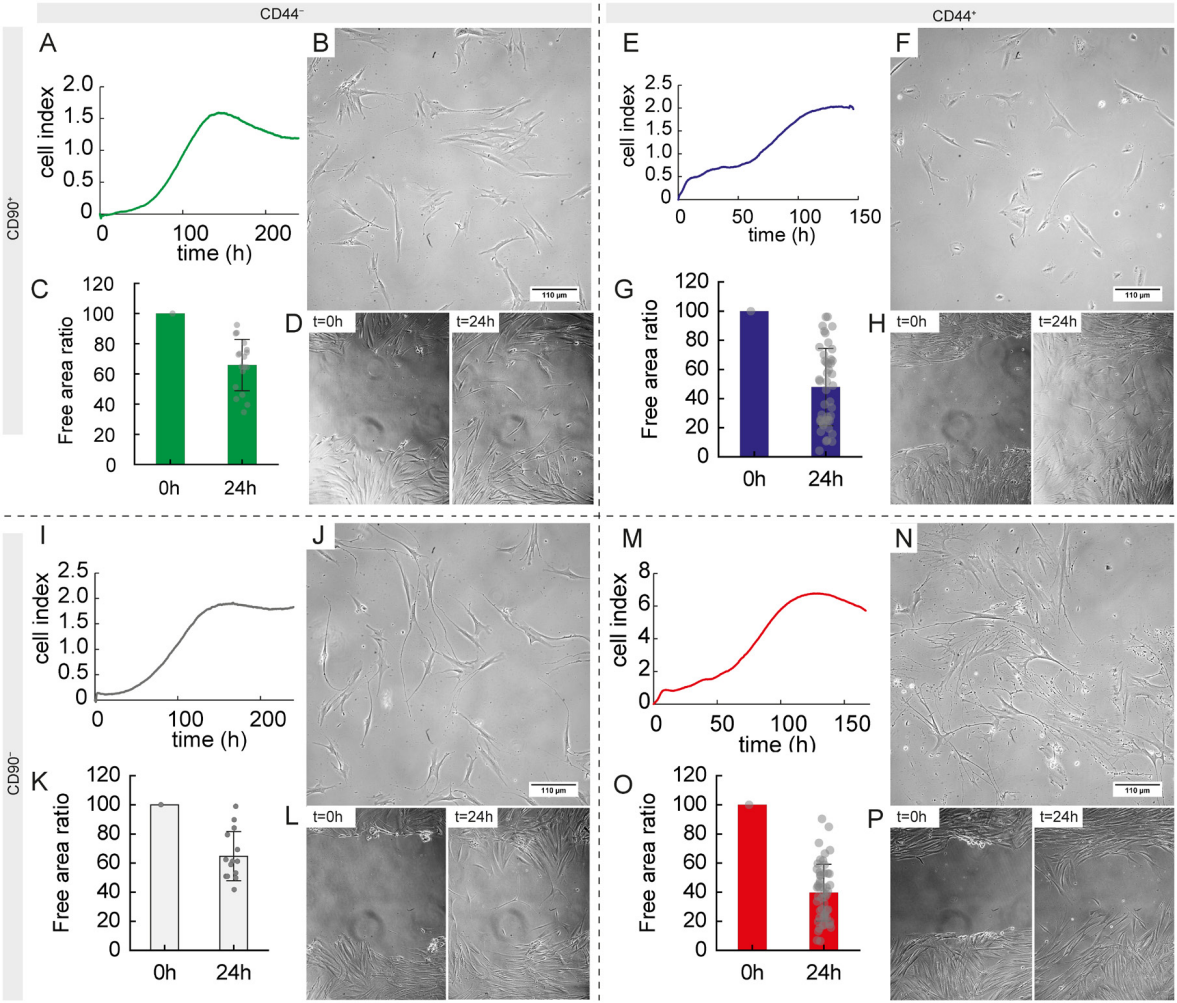
Morphological, growth and migratory characteristics of separated subpopulations according to CD44/CD90 features **(A, E, I, M)** Growth profile using real-time cell analyser. **(B, F, J, N)** Phase contrast microscopy of separated subpopulations, 20× magnification. **(C, G, K, O)** Wound healing (scratch) assay, percentage of free area (higher value mean slower migratory potential and thus lower invasiveness). **(D, H, L, P)** Representative phase contrast images of wound healing assay used for analysis. 10× magnification A–D, CD44^−^/CD90^+^ subpopulation, E–H, CD44^+^/CD90^+^ subpopulation, I–L, CD44^−^/CD90^−^ subpopulation, M–P, CD44^+^/CD90^−^ subpopulation.

The growth curve for CD44^−^/CD90^+^ subpopulation was as follows: lag phase 80 h, exponential phase 37 h, and plateau phase after the 117th h. As soon as cells proceeded into the exponential growth during the exponential phase, the doubling time was measured, because the population is most uniform and cell viability is high in this phase. The doubling time was 26 h (Figure 4A). Morphology of this subpopulation is shown in the Figure 4B. The growth curve for CD44^+^/CD90^+^ subpopulation was as follows: lag phase 74 h, exponential phase 20 h, and plateau phase after the 94th hour. The doubling time was 30 h (Figure 4E). Morphology of this subpopulation is shown in the Figure 4F. The growth curve for CD44^−^/CD90^−^ subpopulation was as follows: lag phase 80 h, exponential phase 40 h, and plateau phase after the 120th hour. The doubling time was 34 h (Figure 4I). Morphology of this subpopulation is shown in the Figure 4J. The growth curve for CD44^+^/CD90^−^ subpopulation was as follows: lag phase 68 h, exponential phase 25 h, and plateau phase after the 92nd h. The doubling time was 30 h (Figure 4M). Morphology of this subpopulation is shown in the Figure 4N.

In the next step, migratory capacity of subpopulations was assessed using a wound healing assay after 24 h. Using univariate testing, CD44 status affected the migratory potential significantly, where cells positive for this antigen demonstrated 1.5-fold higher migratory potential, F(1, 123) = 23.46; p < 0.001. CD90 status did not affected the migratory capacity significantly, F(1, 123) = 1.10, p = 0.29. Nevertheless, CD44^*+*^/CD90^−^ cells exhibited the strongest migratory capacity closely followed by CD44^+^/CD90^+^ subpopulation (1.2-fold lower, p = 0.67); see Figure 4C, 4D, 4G, 4H, 4K, 4L, 4O, 4P.

### Characterization of basal cell death in subpopulations

Double-staining with fluorescein isothiocyanate (FITC)/propidium iodide (PI) was undertaken to determine basal levels of apoptosis and necrosis in particular subpopulations. First, non-stained cells (control) were analysed using flow-cytometry to set the annexin V /PI gating regions (Supplementary Appendix 1A). Consequently, non-treated cells from different subpopulations were analysed (Supplementary Appendix 1B). Four different phenotypes were distinguished: (a) annexin V−/PI− (lower left quadrant, Q3); (b) annexin V+/PI− (lower right quadrant, Q4, usually presumed as apoptotic); (c) annexin V−/PI+ (upper left quadrant, Q1); (d) annexin V+/PI+ (upper right quadrant, Q2, usually presumed as necrotic).

Average frequency of annexin V+/PI− cells was as follows: 14.9% in CD44^−^/CD90^+^ subpopulation; 23% in CD44^+^/CD90^+^ subpopulation; 12.3% in CD44^+^/CD90^−^ subpopulation; 12.6% in CD44^−^/CD90^−^ subpopulation. In sum, frequency of annexin V+/PI− cells in CD90^−^ subpopulations was lower than in CD90^+^ ones.

Furthermore, autophagosome formation in subpopulations was detected using the CYTO-ID Autophagy Detection Kit (Supplementary Appendix 1C). The levels of autophagy (CYTO-ID^+^ population) in subpopulations were as follows: 6.2% in CD44^−^/CD90^+^ subpopulation; 7.8% in CD44^+^/CD90^+^subpopulation; 7.9% in CD44^+^/CD90^−^ subpopulation; 5.5% in CD44^−^/CD90^−^subpopulation. In sum, the frequency of autophagy in CD44^+^ subpopulations was slightly higher than in CD44^−^ ones.

### Expression patterns of HNSCC marker genes in CD44^+^/CD90^+^; CD44^−^/CD90^+^; CD44^+^/CD90^−^; CD44^−^/CD90^−^ subpopulations

This part of study is focused on the expression of genes potentially important for the development of cancer: 1) acquisition of autonomous proliferative signalling (*EGFR, EGF*); 2) proliferative activity of tumor cells (*MKI67*); 3) cell cycle and cell death modifications (*BCL2, BAX, FOS, JUN, TP53, BIRC5, MAP1LC3B, BECN1, NFKB1, MTOR, CAV1*); 4) angiogenesis (*VEGF/FLT1*); 5) metastatic potential (*MMP2*); 6) oxidative stress response (*MT1A, MT2A, ZIP1, HIF1A*), pluripotency genes (*NANOG, SOX2, POU5F1*); and 7) immune response (*CCL2, IL6, IL6R*).

**Figure 5:**
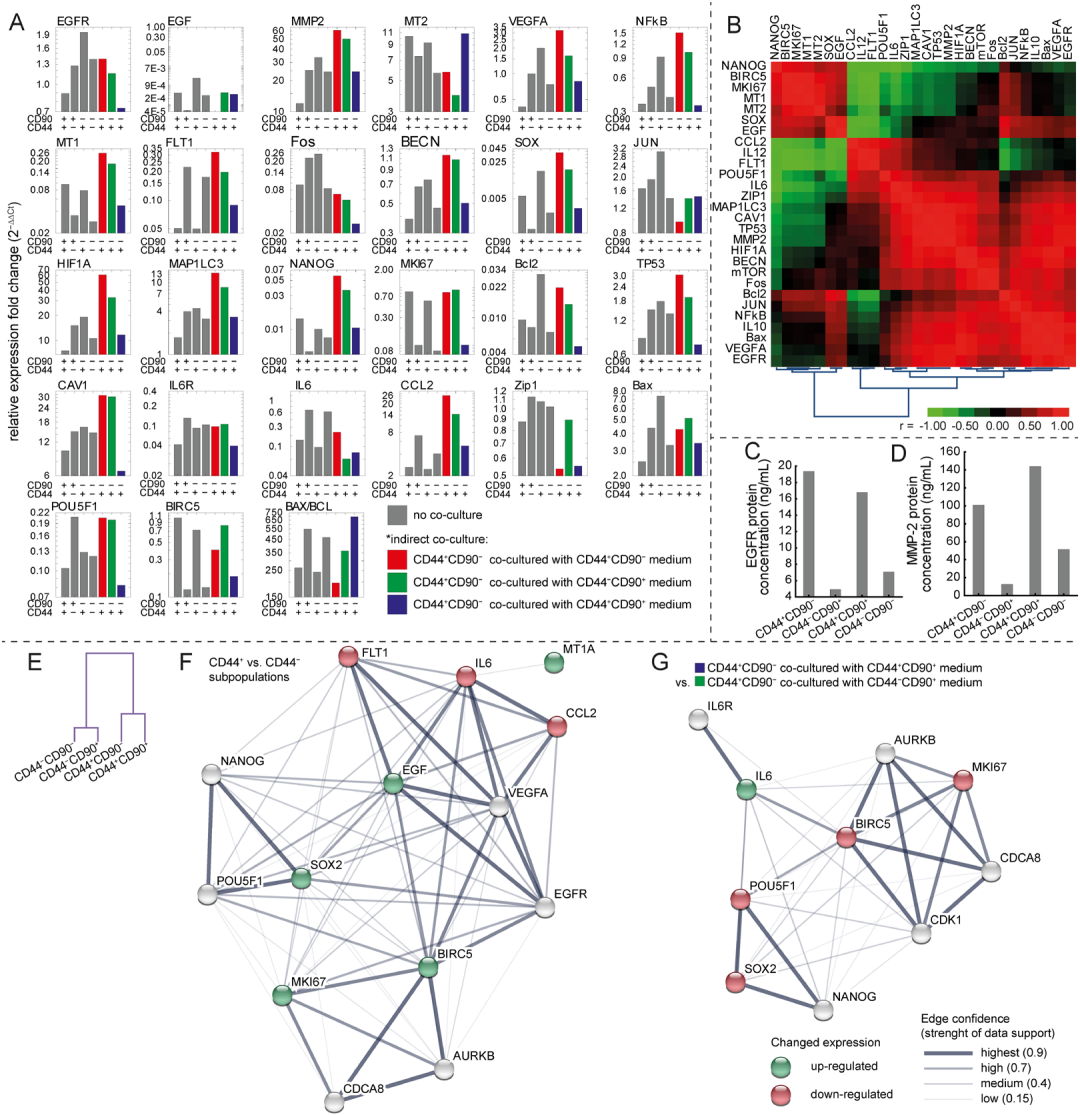
Gene expression in subpopulations and in co-culture experiment **(A)** Gene expression using qRT-PCR. Gray bars indicate measurements without any type of co-culture, coloured bars indicate measurement of gene expression of CD44^+^/CD90^−^ subpopulation affected by medium from particular subpopulation (for details see Materials and Methods section). **(B)** Clustered correlation heatmap based on a gene expression of subpopulations not exposed to co-culture experiment. **(C)** ELISA of EGFR in particular subpopulations. **(D)** ELISA of MMP-2 in particular subpopulations. **(E)** Hierarchical clustering of cases (subpopulations) based on the gene expression, no co-culture only. See the substantial effect of CD44 status on the gene expression. **(F)** Interactome network showing the genes, which expression differs significantly between CD44^+^ vs CD44^−^ subpopulations (green and red for up-, and down-regulation), analyzed using STRING software (version 10.0). Line thickness indicate strenght of data support. **(G)** Interactome network showing the genes, which expression differs significantly between CD44^+^CD90^−^ co-cultured with CD44^+^CD90^+^ medium and CD44^+^CD90^−^ co-cultured with CD44^−^CD90^+^ medium (groups coded blue and green at Figure 5A). For detailed statistical results, see Supplementary Appendix 2, for functional enrichments in the network of selected genes, see Supplementary Appendix 3.

Abundantly expressed genes in all studied subpopulations were *MMP2, HIF1A*, *MT2A*, and *CAV1*. Subpopulation expressing CD44 had significantly higher expression of *BIRC5* and *SOX2* (CD44^+^/CD90^+^ vs. CD44^−^/CD90^+^ p=0.002 resp. p=0.017; CD44^+^/CD90^−^ vs. CD44^−^/CD90^+^ p=0.009 resp. p=0.006). No significant changes in expression between CD44^−^/CD90^+^ and CD44^−^/CD90^−^ or CD44^+^/CD90^−^ and CD44^+^/CD90^+^ were found. Thus, CD90 status did not affect the expression of studied genes significantly (see Supplemantary Appendix 2). On the contrary, CD44^+^ subpopulations had lower expression of *FLT1* and *IL6* compared to CD44^−^ subpopulations (CD44^+^/CD90^+^ vs. CD44^−^/CD90^+^ p=0.03 resp. p=0.0001; CD44^+^/CD90^−^ vs. CD44^−^/CD90^+^ p=0.006 resp. 0.0001), No significant changes in expression between CD44^−^/CD90^+^ and CD44^−^/CD90^−^ were found (see the Figure 5A).

Based on the co-expression pattern of genes, hierarchical clustering revealed that there are two major clusters of subpopulations based on the CD44 status (Figure 5E). Nearness of CD44^+^ subpopulations in gene expression is clearly highlighted, while CD90 status did not affect the overall expression pattern substantially. Subsequently, interactome network showing the genes whose expression differs significantly between CD44^+^ vs CD44^−^ subpopulations was performed using STRING-DB software (Figure 5F). Based on this interactome network, it was revealed that biological processes relating to proliferation, migration, stemness, and angiogenesis were significantly affected by differentially expressed set of genes, (e.g GoMiner GO.0030335, GO.0050678, GO.0001525, GO.0022402, GO.0048646, GO.0016477). For the full list of significantly affected pathways and cellular components see Supplementary Appendix 3.

According to the gene expression correlation analysis (see the Figure 5B), the proliferation marker *MKI67* was in no or even in a negative correlation with proliferative stimuli such as *IL6*, *VEGFA*, or *CCL2.* Aditionally, the expression of receptors such as *EGFR* and *FLT1* was not in a significant positive correlation with their ligands (*EGF*, *IL6*, and *VEGFA*). This implies that part of these factors could be exploited by the subpopulation in tumor mass which is not responsible for their production. For these reasons, experiments with co-cultivation were performed.

Furthermore, protein expression of MMP-2 and EGFR was assessed by ELISA (see Figure 5C and 5D). According to ELISA, CD44^+^ subpopulations had higher levels of both tested proteins compared to CD44^−^. The lowest expression of both MMP-2 and EGFR was detected in CD44^−^CD90^+^ subpopulation of cells.

### Direct co-culture

**Figure 6:**
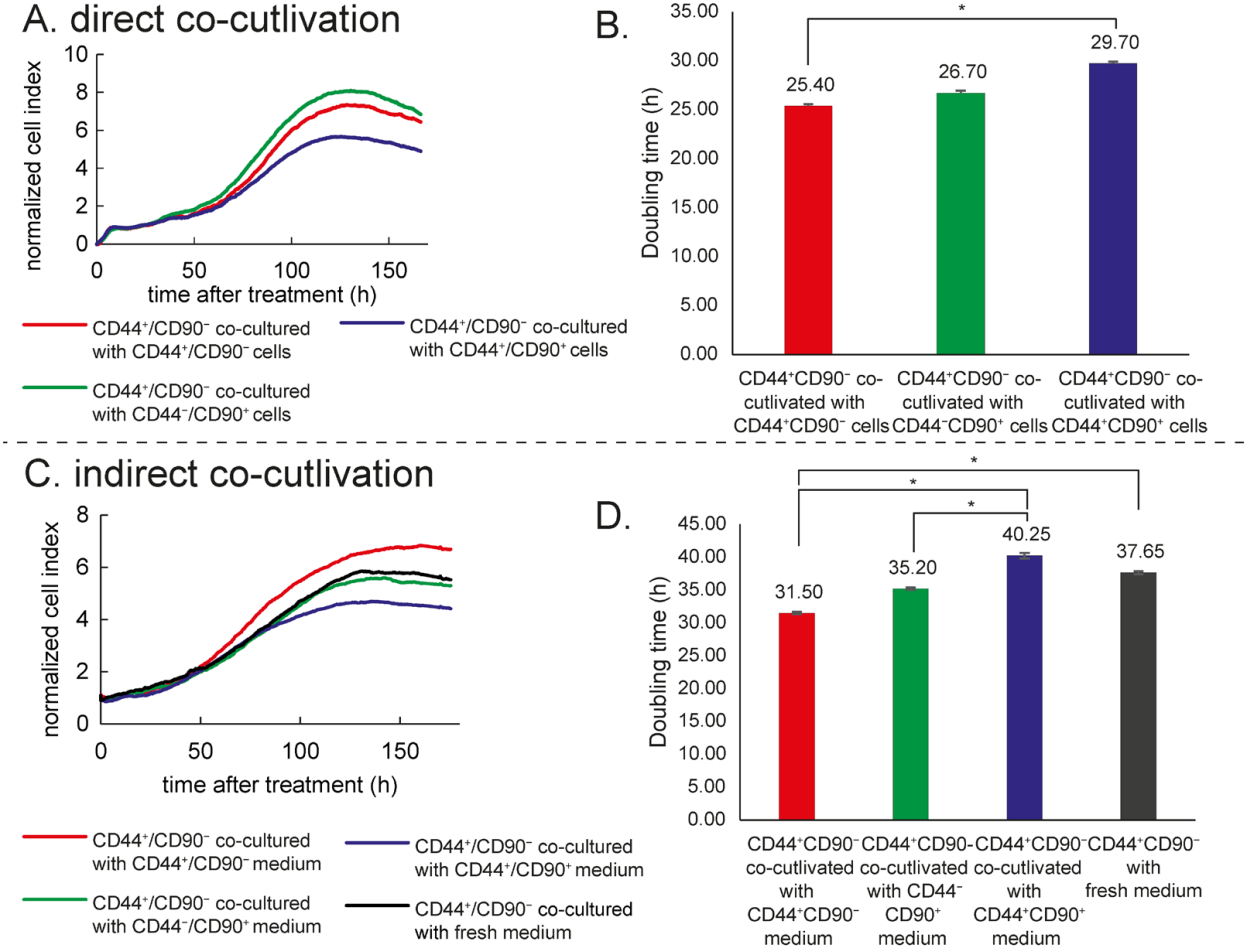
Co-culture experiment **(A)** Direct co-culture (measurement of the CD44^+^/CD90^−^ growth characteristics affected by cells in the insert without a direct contact). Real time cell analyser (RTCA). **(B)** Doubling time of CD44^+^/CD90^−^ cells affected by particular subpopulation in a direct co-culture experiment. **(C)** Indirect co-culture (measurement of the CD44^+^/CD90^−^ growth characteristics affected by a medium from particular subpopulations in a common RTCA setup). **(D)** Doubling time of CD44^+^/CD90^−^ affected by indirect co-culture. Asterisk indicate significant difference at p < 0.05.

After 24 h of incubation, the inserts (containing CD44^+^/CD90^−^; CD44^+^/CD90^+^; or CD44^−^/CD90^+^ cells) were lowered into the E-plate (containing CD44^+^/CD90^−^ cells; anticipated as epithelial tumor cells). In this arrangement, two tested subpopulations were able to exchange factors released to medium and to influence each other's growth by paracrine signaling, but were not able to be in a direct contact. The grow response curve of the “lower” CD44^+^/CD90^−^ population was recorded. The rate of proliferation was monitored in real time using xCELLigence system (see Figure 6A). CD44^−^/CD90^−^ cells were not included in co-cultivation experiments, because they were not able to grow in inserts. Nevertheless, according to our expression and migration analysis, no significant differences between CD44^−^/CD90^−^ and CD44^−^/CD90^+^ subpopulations were observed.

Doubling time of CD44^+^/CD90^−^ cells co-cultivated with CD44^+^/CD90^−^ cells was significantly shorter than doubling time of CD44^+^/CD90^−^ cells co-cultivated with CD44^+^/CD90^+^ (25.40 ± 0.16 h vs. 29.70 ± 0.20 h, p = 0.028); see Figure 6B. Doubling time after co-cultivation with CD44−/CD90+ was shorter as well, but below the statistical significance (26.70 ± 0.22 h, p = 0.06). Doubling time of CD44^+^/CD90^−^ cells cultivated separately without any co-culture was 30 h; (see the section “Morphology, growth and migratory characteristics of subpopulations”). Furthermore, the highest cell index was reached by co-cultivation of CD44^+^/CD90^−^ cells with CD44^−^/CD90^+^. On the contrary, when CD44^+^/CD90^−^ were cells co-cultivated with CD44^+^/CD90^+^, cell index (CI) value was the lowest. This dimensionless parameter is derived as a relative change in measured electrical impedance to represent cell status. When cells are not present or are not well-adhered to the electrodes, the CI is zero. Under the same physiological conditions, when more cells are attached to the electrodes, the CI values are larger. According to our results, factors released by mesenchymal type of cells, which do not express CD44 (CD44^−^/CD90^+^), are able to support a growth of CD44^+^/CD90^−^ type of cells (epithelial tumor cells). Moreover, CD44^+^/CD90^−^ cells also produce factors supporting their own growth.

### Indirect co-culture with subpopulation-derived media

After 24 h-lasting cultivation of CD44^+^/CD90^−^ cells, medium was removed and replaced with a “foreign” media derived from another 24 h-lasting cultivation to display the effect of: a) CD44^−^/CD90^+^ derived medium, b) CD44^+^/CD90^+^ derived medium, c) CD44^+^/CD90^−^ derived medium from different Petri dish, and the effect of d) fresh control RPMI medium. In this arrangement, tested subpopulation was affected by factors released to the medium by other subpopulations (in this arrangement, factors released to medium were not a result of paracrine communication between different cell subtypes like in a direct co-culture experiment). An effect of exhausting of tested medium was also observed. The rate of proliferation was monitored in real time using xCELLigence system (see Figure 6C and 6D). Doubling times of CD44^+^/CD90^−^ cells were as follows: a) 35.20 ± 0.20 h with CD44^−^/CD90^+^-derived medium, b) 40.25 ± 0.41 h with CD44^+^/CD90^+^-derived medium, c) 31.5 ± 0.17 h with CD44^+^/CD90^−^-derived medium from different Petri dish, and d) 37.65 ± 0.22 h with fresh RPMI medium. Thus, cells supplemented with CD44^+^/CD90^+^ growed significantly slower compared to those supplemented with CD44^+^/CD90^−^ medium (p=0.004) and those supplemented with CD44^−^/CD90^+^-derived medium (p=0.030). Furthermore, the highest CI was reached by cultivation with CD44^+^/CD90^−^ derived medium. CI for CD44^−^/CD90^+^ derived medium, and fresh RPMI medium was almost identical. The lowest CI was observed by cultivation with CD44^+^/CD90^+^ derived medium. Inasmuch as tested medium was used for 24 h by other cell population, influence of released factors and nutrient exhausting could be presumed. Whereas CD44^+^/CD90^−^ derived medium from different Petri dish with CD44^+^/CD90^−^ population supported CD44^+^/CD90^−^ growth, CD44^+^/CD90^+^ derived medium rather inhibited CD44^+^/CD90^−^ cell growth in comparison with fresh medium.

Our qRT-PCR measurement has been performed after 72 h of CD44^+^/CD90^−^ cells’ cultivation in “foreign” medium in medium derived from CD44^+^/CD90^−^ cells previously cultivated 24 h in a different Petri dish.

Exhausted medium derived from CD44^+^/CD90^−^ cells caused these particular changes in gene expression in comparison with CD44^+^/CD90^−^ cells in fresh medium (see Figure 5A): (a) Upregulation in *FLT1* (5.15 fold change, p=0.013), *NANOG* (4.58 fold change, p=0.034), *CCL2* (4.81 fold change, p = 0.039), and *IL6* (1.85 fold change, p=0.0001), (b) downregulation in *FOS* (0.25 fold change, p=0.024).

Medium derived from CD44^+^/CD90^+^ caused significant downregulation in expression of *SOX2* and *IL6* (p=0.01 resp. 0.0001) in CD44^+^/CD90^−^ cells (anticipated epithelial tumor cells) compared with medium derived from other CD44^+^/CD90^−^ cultivated separately (see Figure 5A).

Medium derived form CD44^−^/CD90^+^ caused significant downregulation in expression of *IL6* (p=0.0001) in CD44^+^/CD90^−^ cells (anticipated epithelial tumor cells) compared with medium derived from CD44^+^/CD90^−^ (see Figure 5A).

In conclusion, both tested media derived from mesenchymal subpopulations (CD44^+^/CD90^+^ and CD44^−^/CD90^+^) were able to decrease expression of *IL6* in CD44^+^/CD90^−^ cells in comparison with exhausted medium derived from CD44^+^/CD90^−^ cells. The effects on CD44^+^/CD90^−^ cells after a treatment with medium derived from CD44^+^/CD90^+^ cells differed significantly (was either higher or lower) when compared with other two types of partially exhausted subpopulation-derived media (compare Figure 5A). For instance, expression of proliferative marker *MKI67* triggered by tested medium was almost identical to cultivation with CD44^+^/CD90^−^ derived medium, CD44^−^/CD90^+^ derived medium, and fresh RPMI medium, which is in accordance with results obtained from xCELLigence system. CD44^+^/CD90^+^ derived medium rather inhibited *MKI67* expression in CD44^+^/CD90^−^ cells (medium derived from CD44^+^/CD90^+^ caused 0.23 fold change in *MKI67* expression in CD44^+^/CD90^−^ cells in comparison with medium derived from CD44^−^/CD90^+^; p=0.049). Furthermore, *SOX2* and *POU5F1* were also significantly down-regulated. Subsequently, interactome network showing the genes whose expression differs significantly between CD44^+^/CD90^−^ cells co-cultured with CD44^+^/CD90^+^ medium vs. CD44^+^/CD90^−^ co-cultured with CD44^−^/CD90^+^ medium was performed using STRING-DB software (Figure 5G). Based on this interactome network, it was revealed that biological processes relating to the cell division, cell cycle, and cellular response to interleukin-6 were significantly affected by differentially expressed set of genes, (e.g GoMiner GO.0051301, GO.0022402, GO.0071354). For the full list of significantly affected pathways and cellular components see Supplementary Appendix 3.

### Viability after cisplatin treatment

**Figure 7:**
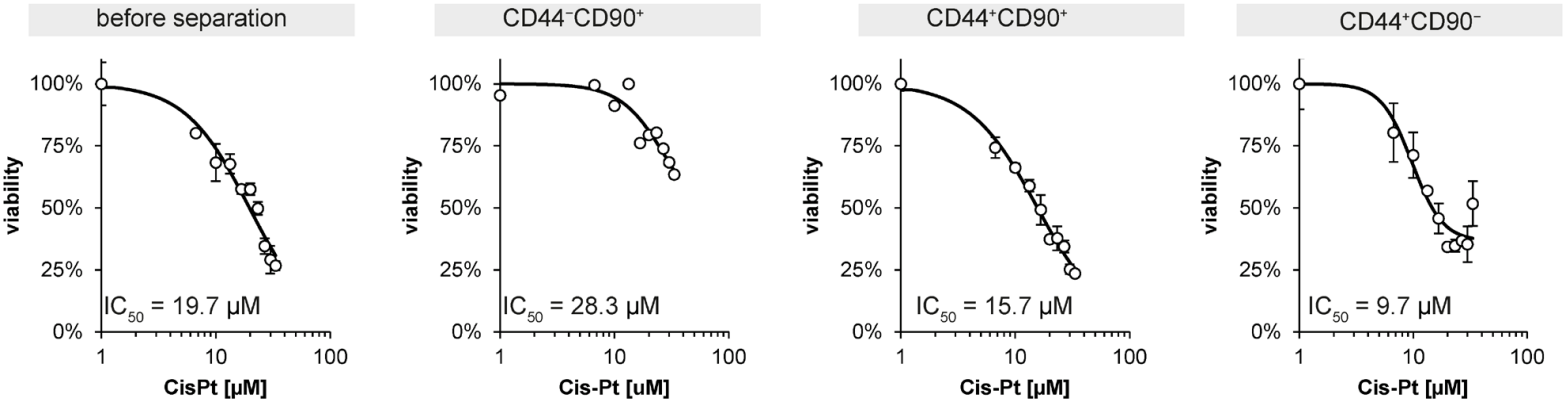
MTT assay of unsorted primoculture and subpopulations exposed to cisplatin treatment IC_50_ means the half maximal inhibitory concentration.

Viability of the primary cell line and the separate subpopulations after cisplatin treatment was tested by using MTT assay in order to assess the degree of resistance of individual subpopulation to this drug. The IC_50_ of primary cell line 132P1 for cisplatin was 19.7 μM. The most resistant subpopulation was CD44^−^/CD90^+^ (IC50 = 28.3 μM). Among CD44^+^ subpopulations, CD44^+^/CD90^+^ was more resistant to cisplatin than CD44^+^/CD90^−^ (IC_50_ value was 15.7 μM and 9.7 μM, respectively), see Figure 7.

## DISCUSSION

In this study, we described the establishment of the HNSCC primary cell line designated 132P1 derived from well-differentiated, localized oral squamous cell carcinoma tissue obtained by the primary tumor biopsy and also establishment and characterization of CD44^+^/CD90^−^, CD44^−^/CD90^−^, CD44^+^/CD90^+^, and CD44^−^/CD90^−^ cell subpopulations derived from this primary cell line 132P1. Also of note, patient was a HPV18-positive, non-smoker, did not receive preoperative radiotherapy or chemotherapy and achieved a complete remission after surgery. Primary cell line prepared by using trypsin proteolysis was more viable than the one prepared by using collagenase (verified also in further primary cell lines preparations; unpublished results). In our established non-separated primary cell line, other subpopulations were gradually overgrown by mesenchymal (CD90^+^ cells) and therefore non-separated HNSCC tissue-derived cell lines are probably not a good model for testing of treatment response of epithelial cancer cells.

Magnetic bead-based separation was found to be suitable for separation of cells with distinct CD-features. All four types of subpopulations were able to grow long-term in cell culture. Nevertheless, after 20th passage, CD44^−^/CD90^−^ and CD44^−^/CD90^+^ subpopulations extremely slowed down their growth and nearly stopped dividing, which was not observed in CD44^+^ subpopulations. It implies that CD44^+^ cells could be more susceptible to immortalization and therefore more predisposed to establishment of permanent HNSCC cell lines. In accordance with our findings, Pries et al. found CD44 to be constitutively expressed on the surface of eight out of eight tested permanent HNSCC cell lines [21]. The doubling time of our primary cell line and derived subpopulations was rather fast (22–34 h) compared to other human tumor cell lines [22].

There is a growing number of studies that confirm a function of cancer-associated stroma in the carcinogenesis and tumor progression. Zhao et al. findings suggest that the phenotype of cells expressing high levels of CD90 is more tumor-promoting than the phenotype of cells expressing low CD90 [23]. According to our results, factors released by CD90^+^ type of cells, which do not express CD44, are able to support growth of epithelial tumor cells (CD44^+^/CD90^−^). On the other hand, factors released by CD44^+^/CD90^+^ cells into medium seem to have rather inhibitory effect on epithelial tumor cell growth. Inasmuch the frequency of annexin V+/PI− and also annexin V+/PI+ cells in CD44^+^/CD90^+^ subpopulation cultivated separately was higher than in other subpopulations, it is possible that CD44^+^/CD90^+^ cells are able to produce factors stimulating cell death in neighbouring cells. This observation is not in accordance with Spaeth et al. who showed that CD44 expression on tumor stromal precursors is necessary for their functionality within the tumor microenvironment as tumor supporting, angiogenesis inducing, activated fibroblasts [24]. Taking into account that our patient achieved a complete remission, we can speculate that the maintenance of tumor is a result of interactions in the cancer cell community and that tumor progression depends on the cooperation among all the members of tumor tissue, not just on the aggressiveness of its most abnormal subpopulation.

In our study, self-survival and self-renewal supporting factors such as *SOX2*, *NANOG*, and *BIRC5* [25–27] were highly expressed in CD44^+^/CD90^+^ subpopulation whereas CD44^−^/CD90^+^ subpopulation was an important producer of “public goods” such as chemokine (C-C motif) ligand 2 (*CCL2*) and interleukin-6 (*IL6*). IL-6 has a possible function in the inducible generation of CSCs and their dynamic balance with non-stem cells [20, 28]. CCL2 supports recruiting of inflammatory monocytes and facilitates metastasis [29]. Furthermore, IL6 and CCL2 are able to support tumor growth, EMT, stem cell migration, and finally treatment resistance [30–34]. Accordingly, we observed the highest cisplatin resistance in CD44^−^/CD90^+^ subpopulation. This subpopulation was more cisplatin-resistant than our primary unseparated cell line 132P1, which implies important role of this subpopulation in conferring of resistance in whole HNSCC tumor.

The most expressed gene in all subpopulations was matrix metalloproteinase 2 (*MMP2*). MMPs promote tumor progression and metastasis formation by degradation of the extracellular matrix. Dufour et al showed that transfection of COS-1 cells with MMP-9, MMP-2, or even with a proteolytically inactive mutant of MMP-9 increases cell migration [35, 36]. ProMMP-2 was also shown to induce vascular endothelial growth factor (VEGF) expression via activation of PI3K/Akt/HIF-1α, which may lead to an increased angiogenesis [37]. Other highly expressed genes in all types of subpopulations were *HIF1A*, *MT2A*, and *CAV1. HIF1A* is known to be involved in hypoxia-induced therapeutic resistance [38], *MT2A* that has been connected with oxidative stress [39], and *CAV1* that has been demonstrated to play an important role in EMT, glucose uptake, lactate accumulation, and ATP production [40–42].

CD44^+^ subpopulations were characteristic by higher gene expression of *BIRC5*, and *SOX2*, protein expression of MMP2 and EGFR and by high basal autophagy. On the contrary, CD44^+^ subpopulations had lower expression of *FLT1* and *IL6*. BIRC alias survivin is involved *inter alia* in the ability to escape from accelerated senescence [43]. Of note, it is possible that senescence-escaped cells may transform into malignant cancer cells by the additional hits of several genes *in vivo*. Elevated levels of autophagy under basal conditions were observed in pancreatic cancer primary tumors and cell lines. Basal autophagy was shown to act as a cellular energy source and to prevent the accumulation of genotoxic levels of oxidative stress in pancreatic cancer cells. Conversely, inhibition of basal autophagy resulted in tumor regression [44]. Furthermore, the expression of *SOX2* gene connected with pluripotency [45] was abundant in both CD44^+^ subpopulations. It could mean that more than one subpopulation with stem cell abilities may exist in the same tumor (CD44^+^/CD90^−^ epithelial cancer stem cells [46] and CD44^+^/CD90^+^ tumor stem cells formed by EMT). Surprisingly, another studied pluripotency gene *POU5F* was abundantly expressed in the CD44^−^/CD90^+^ subpopulation. Mitchell et al. have recently showed that POU5F-induced plasticity in human fibroblasts results in a capability of responding to changes in the extracellular environment that could ultimately lead to the alteration of cell fate. This molecular state of human fibroblasts’ plasticity was characterized by elevated levels of developmental genes, but not other genes involved in pluripotency [47]. We can speculate that *POU5F* expression in non-stem CD44^−^/CD90^+^ subpopulation demonstrates a “willingness” of this subpopulation to be manipulated by aggressive tumor cells.

Radiotherapy represents the standard treatment for head and neck squamous cell carcinoma (HNSCC) patients, often in combination with surgery. Nowadays, chemoradiotherapy has been incorporated in the treatment of advanced tumors. Cisplatin is the most common agent [11]. Based on the doubling time assessment, CD44^−^/CD90^+^ cells proliferated more rapidly than other cells and thus, they might be preferentially eliminated by cisplatin therapy. Nevertheless, the CD44^−^/CD90^+^ subpopulation showed the lowest sensitivity to cisplatin compared to other subpopulations and even compared to the primary cell line. This could be associated with a high expression of *FLT1* by this subpopulation (see Figure 5A) inasmuch as FLT1 kinase was shown as a mediator of resistance in HNSCC [48]. Surprisingly, IC50 values for CD44^+^ subpopulations were lower than IC50 of primary cell line from which subpopulations were derived. This fact could point out on the important role of CD44-negative cells in the development of resistance; support of CD44^+^ by CD44^−^ may be one of the examples. 4 In conclusion, we developed methodics for successful establishement of primary cell lines derived from oral squamous cell carcinoma tissue samples and methodics for removal of mesenchymal (CD90+) cells from this cell line. Separation according to CD44 expression was also successful. CD90/44 status influenced growth rate, sensitivity to cisplatin, and migratory capacity of particular subpopulations derived from the same tumor. Furthermore, non-separated tumor-derived cell lines contained only mesenchymal types of cell after few passages and therefore they are not good model for testing of treatment response of epithelial cancer cells. According to our results, CD90 separation is a necessary step in preparation of permanent HNSCC-tissue derived cell lines. We also presented an indirect cocultivation as an easy and cheap method for evaluation of factors released by distinct subpopulations in tumor, inasmuch as results obtained from direct and indirect settings were comparable. Furthermore, some interesting characteristics of CD44-positive and CD44-negative subpopulations have arisen from our results. Nevertheless, these data are derived only from one HNSCC patient and need further confirmation.

## MATERIALS AND METHODS

### Primary culture establishment and culture conditions

This study was approved by St. Anne University Hospital Ethics Committee (Brno, Czech Republic) and informed consent was obtained from all subjects. All procedures performed in studies involving human participants were in accordance with the ethical standards of the institutional and/or national research committee and with the 1964 Helsinki declaration and its later amendments or comparable ethical standards. Tissue for establishment of the primary cell line were obtained during surgery of patient with oral squamous cell carcinoma (T2N0M0, stage II, grade I) admitted to the Department of Otolaryngology-Head and Neck Surgery, St. Anne’s University Hospital.

The first part of tumor tissue material obtained at surgery was placed into RNAlater (Ambion, USA), the second part into culture medium (RPMI 1640, Biochrom, USA) with an addition of 1% antibiotic-antimycotic solution (Santa Cruz Biotechnology, Texas), 10 μg/ml gentamycin sulphate (Santa Cruz Biotechnology, Texas) and 10 μg/ml ciprofloxacin (Santa Cruz Biotechnology, Texas) to prevent bacteria, fungi and yeast contamination. Within sterile environment and after rinsing the sample by 70% EtOH (Sigma-Aldrich, Germany), the most viable tissue was selected and any necrotic tissue was discarded. Leavings of EtOH were removed by PBS (Invitrogen, USA) washing. Tissue was mechanically dissociated into small pieces. For proteolysis were used: 1. Trypsin (PAA Laboratories GmbH, Austria) protocol 1 and 2. Collagenase (Sigma-Aldrich, Germany) (protocol 2).

### Protocol 1

The small tissue fragments were added and stirred into sterile PBS (Invitrogen, USA) and centrifuged at 4°C, 2700 rpm for 7 min. The cell pellet was re-suspended into 0.25% trypsin in RPMI 1640 medium and left overnight at 4°C. Then medium was removed and tissue was incubated at 37°C for 30 minutes. The cell pellet was re-suspended in medium with an addition of antibiotic-antimycotic solution, gentamycin sulphate, ciprofloxacin and 10% FBS. Primary cell lines were cultivated at 37°C and 5% CO_2_ in humidified atmosphere up to 50% confluence. As soon as cells were seen attaching to the flask surface, medium was changed. Tumor cells were no longer affected by the use of antibiotic-antimycotic solution, gentamycin sulphate, or ciprofloxacin that were added to the early culture. At this time, cells were grown only in Pen/Strep antibiotic solution (PAA Laboratories GmbH, Austria) in complete medium (penicillin 100 U ml^−1^ and streptomycin 0.1 mg ml^−1^; RPMI-1640 medium with 10% FBS (Biochrom, USA)) (Figure 1).

### Protocol 2

The small tissue fragments were added and stirred into sterile PBS and centrifuged at 4°C, 2700 rpm for 7 min. The cell pellet was resuspended into 4.5 ml of complete culture medium with 0.5 ml (2000U/ml) of collagenase. The tissue was incubated at 37°C and 5% CO_2_ for 72 hours and after that tissue was centrifuged at 4°C, 2700 rpm for 7 min. The cell pellet was resuspended in medium with an addition of antibiotic-antimycotic solution, gentamycin sulphate, ciprofloxacin and 10% FBS. Primary cell lines were cultivated at 37°C and 5% CO_2_ in humidified atmosphere up to 50% confluence.

As soon as cells were seen attaching to the flask surface, medium was changed. Tumor cells were no longer affected by the use of antibiotic-antimycotic solution, gentamycin sulphate, or ciprofloxacin that were added to the early culture. At this time, cells were grown only in Pen/Strep antibiotic solution (PAA Laboratories GmbH, Austria) in complete medium (penicillin 100 U ml^−1^ and streptomycin 0.1 mg ml^−1^; RPMI-1640 medium with 10% FBS (Biochrom, USA)) (Figure 1).

### Preparation of subpopulations according to the CD-molecules

Four cell subpopulations were derived from established HNSCC primary cell culture (CD44^+^/CD90^+^; CD44^−^/CD90^+^; CD44^+^/CD90^−^; CD44^−^/CD90^−^). For separation of subpopulation derived from primary cell line magnetic particles- MidiMACS^™^ Starting Kit (CD44 MicroBeads- human, FcR Blocking Reagent-human, LS Columns; Miltenyi Biotec, Germany) and MiniMACS^™^ Starting Kit (CD90 MicroBeads- human, MS Columns; Miltenyi Biotec, Germany) was used.

Dead cells were washed out with 0.5 M EDTA and viable cells were harvested by trypsin. Cell suspension was centrifuged at 300×g for 7 minutes at 4°C. Supernatant was aspirated completely. Cell pelet was resuspended in 1 ml of separating buffer (solution contained phosphate-buffered saline (PBS), pH 7.2, 0.5% bovine serum albumin (BSA), and 2 mM EDTA by diluting MACS^®^ BSA Stock Solution 1:20 with autoMACS^®^ Rinsing Solution). Buffer should be kept in cold (2−8°C). It is important to obtain a single-cell suspension before magnetic labeling. Therefore, cells were passed through 30 μm nylon mesh (Pre-Separation Filters (30 μm), Miltenyi Biotec, Germany) to remove cell clumps. Cell suspension was centrifuged at 300×g for 10 minutes at 4°C. Supernatant was aspirated. Cell pellet was resuspended in 60 μL of buffer and 20 μL of FcR Blocking Reagent per 10^7^ total cells. Then 20 μL of CD44 or CD90 MicroBeads was added. Solution was well mixed and incubated for 15 minutes in the dark in the refrigerator (2−8°C). Cells were washed by adding 1 mL of buffer per 10^7^ cells and centrifugated at 300×g for 10 minutes. Supernatant was aspirated completely. Cell pellet was resuspended in 500 μL of separating buffer. LS or MS column was placed in the magnetic field of a MACS Separator. Column was prepared by rinsing with 3 mL of buffer. Cell suspension was applied onto the column. Flow-through containing unlabeled (CD44 or CD90 non-expressing) cells was collected. Column was washed with 3×3 mL of buffer. Collected unlabeled cells that pass through were combine with the flow–through from previous step and centrifugated at 300×g for 10 minutes. Supernatant was aspirated only 1 mL of buffer was left. These CD44 or CD90 negative cells were then cultivated in RPMI with 10% FBS and ATB. Then column was removed from the separator and was placed on a suitable collection tube. 5 mL of buffer were pipetted onto the column. the magnetically labeled cells were flush out by firmly pushing the plunger into the column. These CD44 or CD90 positive cells were then cultivated in RPMI with 10% FBS and ATB.

Cells that adhered to the flask were grown in complete medium (RPMI-1640 medium with 10% FBS, penicillin 100 U ml^−1^ and streptomycin 0.1 mg ml^−1^) until they reach 70% confluency, they were then passaged.

### CD44 and CD90 fluorescent staining

At the logarithmic growth phase, cells were stained by CD90 and CD44 antibodies to detect their cell surface marker expression. The cells were washed three times with PBE (0.1 PBS containing 0.5% bovine serum albumin and 0.002 M EDTA (PAA Laboratories GmbH, Austria); pH 7.2) and stained with CD44-FITC/CD90-APC according to manufacturer’s instructions (1:400, Miltenyi Biotec, Germany). Nuclei were co-stained with Hoechst 33258 (2 μM, Invitrogen, USA). Nikon Eclipse Ti-S equipped with appropriate set of filters (Japan) was used to visualize the cells. A bar represents 100 μm.

### Cell number quantification

Total cell numbers were analysed using the Casy model TT system (Roche, Switzerland) and the following protocol: first, calibration was performed form viable and necrotic cells. For necrotic cells, 100 μl cell suspension and 800 μl Casy Blue solution was mixed and left for 10 min at room temperature. Subsequently, 9 ml Casy Tone was added. To prepare a viable cell standard, 100 μl of cell suspension was mixed with 10 ml of Casy Tone. All subsequent measurements were made in 100× diluted 100 μl cell suspension. Prior to each measurement, background was subtracted. All samples were measured in triplicates.

### MTT viability assay

The MTT assay was used to determine cell viability. The suspension of cells in the growth medium was diluted to a density of 2000–10000 cells/column in 200 μl medium and transferred to wells 2–11 of standard microtiter plates. The medium (200 μl) was added to the first and to the last column (1 and 12). The plates were incubated for 2 days at 37°C to ensure the cell growth. The medium was removed from columns 2 through to 11. Columns 3–10 were filled with 200 μl of the medium containing different concentrations of cisplatin (0–34 μM). As a control, columns 2 and 11 were fed with the medium only. The plates were incubated for 48 h. After that, columns 1–11 were fed with 200 μl of the medium with 50 μl of MTT (5 mg/ml in PBS) and incubated for 4 h in a humidified atmosphere at 37°C, wrapped in the aluminium foil. After that, the medium was exchanged with 200 μl of 99.9% DMSO to dissolve MTT-formazan crystals. Then, 25 μl of glycine buffer was added to all wells with DMSO and the absorbance was recorded at 570 nm (VersaMax microplate reader, USA).

### Real-time impedance based cell growth and proliferation

The impedance-based real-time cell analysis (RTCA) xCELLigence system was used according to the instructions of the supplier (Roche, Switzerland). Firstly, the optimal seeding concentration for proliferation and cytotoxic assay was determined. Optimal response was found for primary cell line (2,000 cells/well) and all subpopulations: CD44^+^/CD90^−^ (2,000 cells/well), CD44^+^/CD90^+^ (1,000 cells/well) CD44^−^/CD90^−^ (5,000 cells/well), CD44^−^/CD90^+^ (2,000 cells/well). After seeding a total number of cells in 200 μl of medium to each well in E-plate 16, the attachment and proliferation of the cells were monitored every 15 min. Duration of all experiments was 150 h. Results are expressed as relative impedance. In all of studied subpopulations doubling time was determined by using manufacturer’s software.

### Preparation of subpopulation-derived media

24 h prior to conducting experiments, CD44^−^/CD90^+^, CD44^+^/CD90^+^ and CD44^+^/CD90^−^ cells were trypsinized. 50,000 cells/ml of each cell line were cultured in 75 cm^2^ flasks with medium. Media were removed from cultures after 24 h and used for the experiments.

### Indirect co-culture

The rate of proliferation was monitored in real time using xCELLigence system (E-plate). 2000 CD44^+^/CD90^−^ cells per well were seeded in the E-plate 16. After 24 h of impedance reading, medium was removed from each well and replaced by media derived from foreign subpopulations (CD44^−^/CD90^+^, CD44^+^/CD90^+^) and by control medium derived from the same subpopulation (CD44^+^/CD90^−^) or by fresh RPMI medium. Impedance value was automatically monitored by the system for 200 h. This experiment was performed in duplicates.

Furthermore, determining of changes in gene expression after incubation with medium derived from foreign subpopulation was performed. The CD44^+^/CD90^−^ cells were cultivated at 37 °C and 5% CO_2_ in humidified atmosphere up to 70% confluence. After 24 h medium was removed and replaced with foreign media: a) CD44^−^/CD90^+^, b) CD44^+^/CD90^+^ and c) by control medium (CD44^+^/CD90^−^). The experiment has been performed for 72 h than the relative expression of 15 genes related to HNSCC pathogenesis was determined by using qRT-PCR in all of these cases.

### Direct co-culture

The rate of proliferation was monitored in real time using xCELLigence system (E-plate and E-insert). 2000 CD44^+^/CD90^−^ cells per well were seeded in the E-plate 16. CD44^+^/CD90^−^, CD44^+^/CD90^+^ and CD44^−^/CD90^+^ cells were seeded into insert at the density of 15000 cells per well. After 24 h of incubation at 37°C and 5% CO_2_ the insert was lowered into the E-plate. Impedance value was automatically monitored by the system for 140 h. This experiment was performed in duplicates.

### ELISA analysis

Levels of EGFR and MMP2 in homogenized cells were determined by commercial enzyme-linked immunosorbent assay (ELISA) kits (RayBiotech, USA) according to the manufacturer’s instructions. The EGFR-ELISA is designed to detect human EGFR with a detection limit 4 pg/ml, a 10% intra-assay, and a 12% inter-assay variability. The EGFR antibodies were raised against the L25–S645 region of EGFR. The MMP2-ELISA is designed to detect human MMP2 with a detection limit 3500 pg/ml, a 10% intra-assay, and a 12% inter-assay variability.

### RNA isolation and reverse transcription

TriPure Isolation Reagent (Roche, Basel, Switzerland) was used for RNA isolation. The isolated RNA was used for cDNA synthesis. RNA (1000 ng) was transcribed using transcriptor first strand cDNA synthesis kit (Roche, Switzerland), which was applied according to manufacturer's instructions. The cDNA (20 μl) prepared from the total RNA was diluted with RNase free water to 100 μl and the amount of 5 μl was directly analysed by using the LightCycler^®^480 II System (Roche, Basel, Switzerland).

### Quantitative real-time polymerase chain reaction

qRT-PCR was performed using the TaqMan gene expression assays with the LightCycler^®^480 II System (Roche, Basel, Switzerland) and the amplified DNA was analysed by the comparative Ct method using β-actin as an endogenous control. The primer and probe sets for ACTB (assay ID: Hs99999903_m1), MT2A (Hs02379661_g1), MT1A (Hs00831826_s1), TP53 (Hs01034249_m1), BAX (Hs00180269_m1), BCL2 (Hs00608023_m1), VEGFA (Hs00900055_m1), FLT1 (Hs01052961_m1), MMP2 (Hs01548727_m1), FOS (Hs00170630_m1), JUN (Hs00277190_s1), MKI67 (Hs00606991_m1), EGF (Hs01099999_m1), EGFR (Hs01076078_m1), SOX2 (Hs01053049_s1), NFkB1 (Hs00765730_m1), BECN1 (Hs00186838_m1), HIF1A (Hs00153153_m1), MAP1LC3B (Hs00797944_s1), NANOG (Hs04260366_g1), CAV1 (Hs00971716_m1), MTOR (Hs00234508_m1), CCL2 (Hs00907239_m1), ZIP1 (also known as SLC39A1) (Hs00205358_m1), BIRC5 (Hs00153353_m1), IL6 (Hs00985639_m1), IL6R (Hs01075666_m1), and POU5F1 (Hs04260367_gH) were selected from TaqMan gene expression assays (Life Technologies, USA). qRT-PCR was performed under the following amplification conditions: total volume of 20 μl, initial incubation at 50°C/2 min followed by denaturation at 95°C/10 min, then 45 cycles at 95°C/ 15 sec and at 60°C/1 min. Gene expression experiments were performed in duplicates

### Wound healing assay

After passage each cell line was resuspended and seeded into 24-well plate, the cell amount per well in 500 μl media was optimized to 60,000 for CD44^+^/CD90^+^, 50,000 for CD44^+^/CD90^−^, 40,000 for CD44^−^/CD90^−^ and 45,000 for CD44^−^/CD90^+^. After 48 h the cells were 100% confluent and the scratch into the cell monolayer was made. After gentle wash and change of media each well was photographed at time 0 and after 24 h at the very same spot. The photos were analysed according to instructions from the software creator [49]. The software computed the percent of open wound area. Each cell line was analysed in at least 24 repetitions.

### Flow cytometric analysis of cell death

Double-staining with fluorescein isothiocyanate (FITC)/propidium iodide (PI) was undertaken using the Annexin V-FLUOS-staining kit (Roche Applied Science) according to the manufacturer’s protocol in order to determine percentages of viable, apoptotic and necrotic cells. Briefly, the cells were harvested by repetitive pipetting and washed two times with PBS (centrifuged at 2000 rpm for 5 min), resuspended in 100 μl of Annexin-V-FLUOS labelling solution and incubated in the dark at 15–25°C (15 min.). Annexin V-FITC fluorescence was detected by flow cytometry (Partec GmbH, Münster, Germany); (FL1 filter for Annexin-V-FLUOS and FL3 filter for PI).

### Flow cytometric detection of autophagosomes

Autophagosome formation in subpopulations were detected using the CYTO-ID Autophagy Detection Kit (Enzo, PA, USA) following the manufacturer’s instruction. The CYTO-ID green fluorescent reagents specifically detect acid autophagic vacuoles formed during autophagy. Briefly, the cells were harvested by gentle repetitive pipetting, spun down and washed twice in RPMI 1640 with 5% fetal bovine serum (FBS), then were centrifuged at 2000 rpm for 5 min. The cells were resuspended in 500 μl of freshly diluted CYTO-ID staining reagent and incubated in the dark at 37°C for 30 min. CYTO-ID fluorescence of cells was immediately analysed by flow cytometry using the flow cytometry (Partec GmbH, Münster, Germany) (FL1 filter for CYTO-ID, SSC for cellular granularity). The percentage of cells with CYTO-ID staining was used to represent the formation of autophagosomes.

### HPV detection

The 142 base-pair long sequence of conservative *L1* gene was amplified using GP5 and GP6 primers for non-specific identification of HPV-positive subjects. The PCR mixture from New England Biolabs (UK), contained PCR buffer (10 mM Tris HCl, pH 8.3, 50 mM KCl with 2.5 mM MgCl_2_ included) 0.05 mM of each dNTP and 0.05 mM of GP5 (5’-TTTGTTACTGTGGTAGATAC-3’) and GP6 (5’-GAAAAATAAACTGTAAATCA-3’) primers. The DNA amplification was carried out during 40 cycles that included the denaturation at 94°C for 30 s, annealing at 45°C for 30 s and the primer extension at 72°C for 30 s.

The HPV-positive specimens were further analysed with the HPV16 (FOR primer: 5’-CCCAGCTGTAATCATGCATGGAGA-3’; REV primer: 5‘-GTGTGCCCATTAACAGGTCTTCCA-3‘) and HPV18 (FOR primer: 5’-CGACAGGAACGACTCCAACGA-3’; REV primer: 5‘-GCTGGTAAATGTTGATGATTAACT-3‘) primers. The PCR amplicons reached length of 202 bp for HPV16 and 272 bp for HPV18. The DNA amplification was carried out during 40 cycles that included the denaturation at 94 °C for 30 s, the annealing at 58°C for 30 s and the primer extension at 72°C for 30 s. As internal quality control of the isolated DNA, β-actin gene (600 bp) was amplified (FOR primer: 5’CCTGAACCCTAAGGCCAACC3’; REV primer: 5’GCAATGCCTGGGTACATGGT3’). Each PCR product was analysed using electrophoresis on 1% agarose gels stained with ethidium bromide.

### Statistical analysis

Pearson correlation and cluster analysis were performed to reveal associations between cases and variables. These analyses were performed on standardized data; the cluster analysis was performed using Ward’s method. Data were analysed using factorial ANOVA following planned comparisons. All charts are depicted with means and standard deviations. P value < 0.05 was considered significant. Software Statistica (StatSoft, Tulsa, OK, USA) was used for analysis. The annotation analyses were performed using the GoMiner (http://discover.nci.nih.gov/gominer/index.jsp), interactome network was constructed using the STRING software (http://string-db.org/) using Kyoto Encyclopedia of Genes and Genomes (KEGG) pathway database (http://www.genome.jp/kegg/), which provides gold standard sets of molecular pathways.

## SUPPLEMENTARY MATERIALS APPENDIX






