# Supplementary material for: Establishment of oral squamous cell carcinoma cell line and magnetic bead-based isolation and characterization of its CD90/CD44 subpopulations
Source: Oncotarget. 2017 Aug 3;8(39):66254–69. doi: 10.18632/oncotarget.19914 (PMC5630409; doi:10.18632/oncotarget.19914)
Supplement: Supplementary file 1 [file oncotarget-08-66254-s001.pdf]

Establishment of oral squamous cell carcinoma cell line and magnetic bead-based isolation and characterization of its CD90/CD44 subpopulations

SUPPLEMENTARY MATERIALS

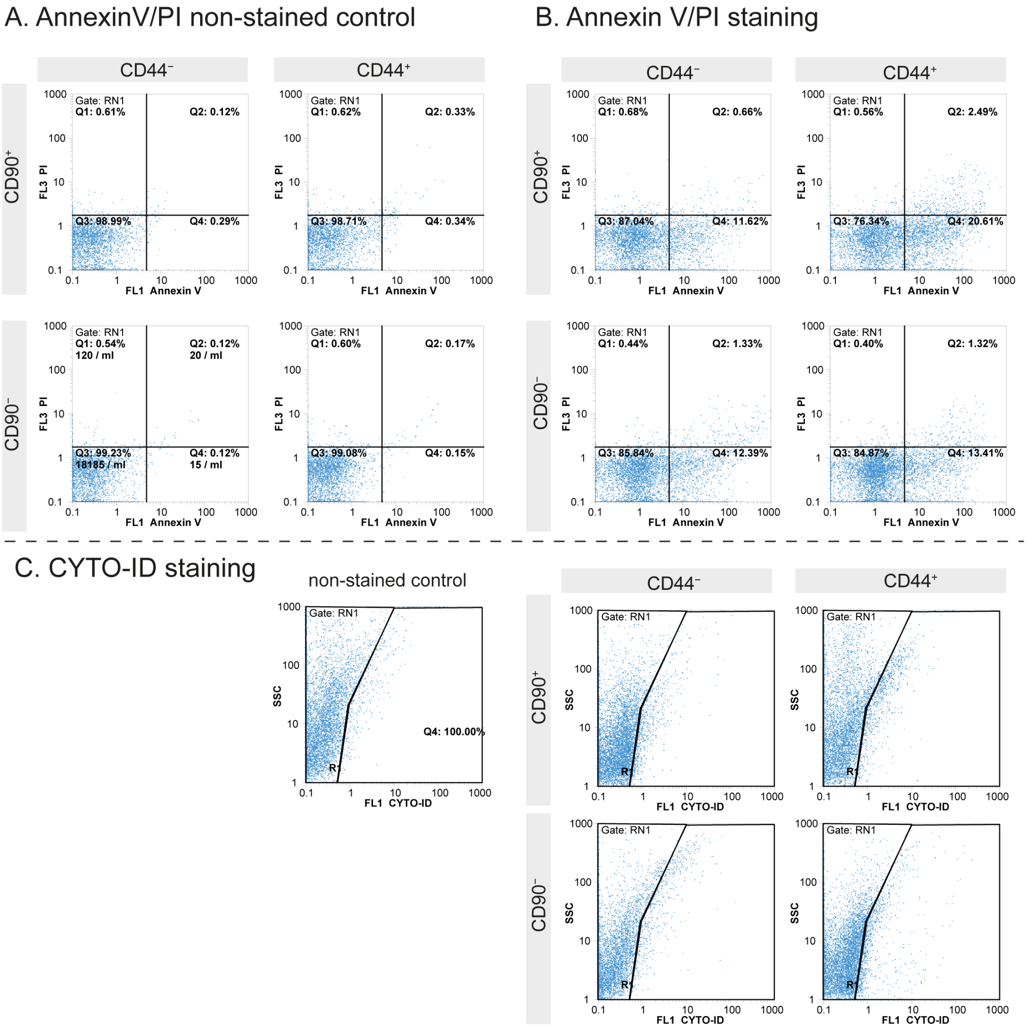

Supplementary Appendix 1: Flow-cytometric analysis of AnnexinV/propidium iodide and CYTO-ID staining of subpopulations. **(A)** Non-stained control for the setting of gating regions. "RN1" indicates exlusion of cell debris based on forward scatter, which is applied for all further gating. **(B)** Annexin V/PI staining. **(C)** CYTO-ID gating for the analysis of autophagic vacuoles. Non-stained control (left) for the setting of the gating regions and four subpopulations (right).

Supplementary Appendix 2: Results of one-way ANOVA. Gene expression data shown as a gene expression fold change, 95% confidence interval and its p-level.

See Supplementary File 1

Supplementary Appendix 3: Functional enrichments in the network of selected genes. Based on GoMiner (GO) and Kyoto Encyclopedia of Genes and Genomes (KEGG) pathway database, performed using the STRING software (http://string-db.org/). Only those functional enrichments showing significant false discovery rate are included. First list of excel describes set of genes differentialy expressed between $CD44^{+}$ and $CD44^{-}$ subpopulations, second list decribe list of genes differentialy expressed between "$CD44^{+}$/$CD90^{-}$ co-cultured with $CD44^{+}$/$CD90^{+}$ medium vs. $CD44^{+}$/$CD90^{-}$ co-cultured with $CD44^{-}$/$CD90^{+}$ medium".

See Supplementary File 2
